# Maternal Death, Autopsy Studies, and Lessons from Pathology

**DOI:** 10.1371/journal.pmed.0050048

**Published:** 2008-02-19

**Authors:** Sebastian Lucas

## Abstract

The author discusses the implications of a new autopsy study of maternal deaths in Mozambique.

Pathology is quality control—it confirms, refutes, or elaborates on clinical diagnoses. Globally, the most important fatal condition for which there is a lack of autopsy evaluation is maternal death. One reason for this lack may be that everyone supposedly knows why mothers die in pregnancy and childbirth. In resource-poor or developing countries, where the World Health Organization (WHO) estimates that over 99% of the 500,000 or more global maternal deaths occur annually, the stated main causes are (in descending order): post-partum haemorrhage, puerperal sepsis, hypertensive disorders, obstructed labour, and abortion. These are the so-called direct conditions, i.e., they would not happen if the woman were not pregnant. But WHO admits that it cannot reliably estimate these, nor the additional morbidity and mortality burden of the indirect conditions that lead to maternal death, i.e., pre-existing diseases that are worsened by pregnancy and delivery. These conditions include HIV, malaria, anaemia, tuberculosis, cardiac disease, and post-partum suicide; globally they are estimated to be less frequent than the direct causes [1].

In the United Kingdom, where maternal deaths are exhaustively evaluated (including through many autopsies), the indirect causes are more common than the direct ones by a ratio of more than 1.2. Overall the causes are (in descending order): maternal cardiac disease (acquired and congenital), pulmonary thromboembolism, central nervous system disorders, hypertensive disorders, sepsis, psychiatric disorder and suicide, haemorrhage, amniotic fluid embolism, and infectious diseases (of which HIV is a small proportion) [2].

A second reason for the global lack of autopsy data relates to the circumstances of maternal death in resource-poor countries. Most deliveries and deaths occur not in hospitals but in rural areas, where there may be no midwives or traditional birth attendants, let alone obstetricians [3]. So as well as being less well counted, maternal deaths will not be regularly evaluated by pathologists.

A third possible reason might be ministerial embarrassment. Whilst road traffic collisions leading to death are routinely examined by autopsy (though the information gained is generally small), there may be less desire to investigate deaths that collectively represent the greatest contrast in health outcomes between poor and rich countries. The lifetime risk of maternal death for a female in north-western Europe is one in 30,000, whilst in Afghanistan and Sierra Leone it is one in six [3]. And in many countries, maternal deaths are partly the outcome of policy and legislation, such as the criminalisation of therapeutic abortion, which makes so many women seek unsafe termination of pregnancy [4].

**Linked Research Article**

This Perspective discusses the following new study published in *PLoS Medicine*:

Menéndez C, Romagosa C, Ismail MR, Carrilho C, Saute F, et al. (2008) An autopsy study of maternal mortality in Mozambique: The contribution of infectious diseases. PLoS Med 5(2): e44. doi:10.1371/journal.pmed.0050044

Menéndez and colleagues conducted a prospective study on the causes of maternal death in a tertiary-level referral hospital in Maputo, Mozambique, using complete autopsies with histological examination.

## A New Autopsy Study of Maternal Deaths in Mozambique

For all the above reasons, a new formal investigative study published in *PLoS Medicine* examining maternal deaths in Mozambique [5], a country that WHO lists as having the fifteenth highest maternal mortality rate (MMR) in the world [1], is welcome. Autopsy studies of maternal deaths in poor countries are rare, and virtually all the countries with MMRs greater than 1,000 out of 100,000 live births are in Africa.

The new study, by Clara Menéndez and colleagues, examines prospectively, by autopsy with full histopathology, 78% of the 179 maternal deaths (WHO definitions) that occurred in the tertiary referral hospital in Maputo over 26 months in 2002–2004, with particular interest in the contributions of HIV and malaria. (Previous studies in Africa have highlighted the contribution of HIV to maternal death, but none have involved systematic autopsy analysis [6].) In Menéndez and colleagues' study, 53% of the women tested were HIV infected. Obstetric complications (direct deaths) accounted for 41% of deaths: haemorrhage, sepsis, and hypertensive disorders made up 87% of these deaths. Infectious diseases not directly related to pregnancy accounted for 48% of all deaths, with HIV-related pathology, pyogenic pneumonia, severe malaria, and pyogenic meningitis the most important causes of death. Overall, 13% of all maternal deaths were due to HIV disease (more than half of these involved tuberculosis), and 10% to malaria. The study demography found that the hospital's MMR was 847 out of 100,000, which was a tripling of the rate a decade earlier.

The conclusions are stark. In contrast to received wisdom, direct maternal deaths were less frequent than indirect ones, with infectious diseases accounting for half of all deaths. Effective treatments for the leading infectious diseases are available, as are preventive measures against malaria and pneumococcal disease. Nonetheless, it should be noted that the overall commonest cause of death was still haemorrhage, which represents a failure of the local health care systems (in terms of measures such as provision of safe blood transfusion and adequate obstetric care) [5].

## Strengths and Limitations of the Study

The study combined a review of the medical records with autopsy and laboratory data, to provide final causes of death in the majority of maternal deaths over a recent two-year period. This was a unique achievement for an African country.

A problem for such studies is correlating the often limited clinical data with objective autopsy and histopathology to come up with a final cause of death. The process can be subjective [7], and thus it is reassuring that 6% of the maternal deaths examined were labelled as “unknown cause” rather than forced artificially into more certain categories. Case definitions are another problem in this type of study. Some might consider “pyogenic pneumonia and meningitis” to fall within the compass of HIV-related disease, which would make HIV disease the commonest cause of death. Sepsis is difficult to identify precisely without ante-mortem and post-mortem blood cultures (these were not available), which can lead to clinical over-estimation of the condition and pathological underestimation, since there are no specific pathological features of sepsis [8]. And is sepsis in those with HIV necessarily worsened by HIV?

The most important limitation is geographical. Most maternal deaths in poor countries happen away from the big hospitals [3], making referral centre data inevitably unrepresentative. Haemorrhage from difficult and obstructed deliveries, with no access to expert assistance, may still be the single most important cause of maternal death. Such women do not make it alive to the referral hospitals.

## Implications and Next Steps

Malaria was carefully defined in the study, and the data will contribute to the debate on the complex associations between maternal death, malaria, and HIV. HIV (particularly with tuberculosis) needs to be considered even more carefully in the management of pregnancy in areas of high HIV endemicity.

We still need a truly representative multi-disciplinary study (i.e., with pathology) of maternal death in poor countries, with and without HIV. Only then will there be a more informed debate on the critical question: where do you put the money if you want to reduce maternal death [9]? Into broad decentralised provision of better (but low-tech) antenatal and midwifery care? Or into hospitals, which have the capacity to intervene quickly and successfully when things go wrong, with the aim of having most or all deliveries eventually take place there? Pathology data here could truly influence national and international policy.

## Abbreviations

MMR: maternal mortality rate
WHO: World Health Organization
